# Diabetes, Fetal Demise, and Shoulder Dystocia: The Importance of Glucose Screening to Prevent Catastrophic Obstetric Outcomes

**DOI:** 10.1155/2020/8142109

**Published:** 2020-03-10

**Authors:** S. Ahmed Hussain, Alisha M. Smith, Jennifer A. Cross

**Affiliations:** Department of Obstetrics and Gynecology, Naval Medical Center Portsmouth, USA

## Abstract

Diabetes is associated with increased risk of stillbirth and shoulder dystocia. Compared with uncomplicated pregnancies, diabetic patients have a 4-6x risk of stillbirth and 2-3x risk of shoulder dystocia. A 34 yo G2P0010 presented with a 40+3 wga IUFD with nonstandard antenatal glucose screening. Admission labs included a hemoglobin A1c of 6.6. She had a vaginal delivery complicated by a 30-minute shoulder dystocia that was not relieved by McRoberts, suprapubic pressure, Rubin II, Wood's Screw, or posterior arm delivery. Nitroglycerine was administered, after which Wood's Screw was successful resulting in delivery of an infant weighing 4190 grams (85th percentile for gestational age). A 31 yo G1 presented with a 37+1 wga IUFD. Her 28 wga three-hour GTT was notable for an elevated value at one hour (216 mg/dL). Admission labs included a hemoglobin A1c of 6.6. She had a vaginal delivery complicated by a 30-minute shoulder dystocia that was relieved via posterior axillary sling after failure of McRoberts, suprapubic pressure, Rubin II, Wood's Screw, and Gaskin's, resulting in the delivery of an infant weighing 3590 g (92nd percentile for gestational age). We present two cases of severe shoulder dystocia in patients who both presented with term IUFD and diabetic-range hemoglobin A1c. There is minimal literature on diabetic patients with pregnancies affected by both stillbirth and shoulder dystocia. These cases underscore the importance of glucose screening and control to prevent catastrophic obstetric outcomes.

## 1. Introduction

In 1909, Williams reported a maternal mortality rate of 54% and perinatal mortality rate of up to 65% in pregnancy complicated by what was described at that time as glycosuria [1]. It is now known that diabetes in pregnancy—whether preexisting or diagnosed during gestation—is associated with significant adverse maternal and perinatal outcomes including preeclampsia, shoulder dystocia, fetal birth injury, neonatal hypoglycemia, macrosomia, and intrauterine fetal demise (IUFD) [2]. Approximately 4% of pregnancies in the United States are complicated by pregestational diabetes, and another 8% are complicated by gestational diabetes [3].

While glycemic control in pregnancy at the population level has improved significantly in the last century as a result of screening and treatment efforts, 4% of stillbirths (defined as fetal demise beyond 20 weeks gestational age) are attributable to diabetes. The stillbirth rate in pregnancies complicated by diabetes is 4-6 times than that of uncomplicated pregnancies [4]. The underlying etiology of fetal demise in infants of diabetic mothers is not identified in up to 50% of cases. One of the most common causes of fetal demise in a pregnancy complicated by diabetes is congenital malformations such as cardiovascular defects or the VACTERL cluster (vertebral anomalies, anal atresia, cardiac defect, trachea-esophageal fistula, renal abnormalities, limb abnormalities) [5]. Other etiologies include diabetic ketoacidosis, which is associated with a perinatal mortality rate of 50-90%, and fetal hyperglycemia and hyperinsulinemia which can both cause fetal hypoxia and subsequent demise [4]. Maternal factors such as preeclampsia and vasculopathy, both of which are associated with diabetes, can also compromise placental blood flow and fetal oxygenation and result in fetal demise.

Maternal diabetes has also been shown to be a risk factor for shoulder dystocia, a potentially devastating birth event that can cause fetal injury or death. Diabetes increases the risk of shoulder dystocia by a factor of six via multiple mechanisms [6]. Infants of diabetic mothers are more likely to be macrosomic (defined as birth weight of greater than or equal to 4000 grams) with an odds ratio of 2.19 when compared to infants of nondiabetic mothers [5]. The incidence of shoulder dystocia amongst macrosomic infants, regardless of diabetic status, is 13% compared to 1% when the birth weight is under 4000 g [7]. It is theorized that other factors of fetal biometry affected by maternal glucose control also factor into the increased rate of shoulder dystocia in infants of diabetic mothers. For example, infants of diabetic mothers have significantly greater shoulder-to-head and chest-to-head proportions than those of nondiabetic mothers [8].

Shoulder dystocia is notoriously difficult to predict, but other classically described risk factors in addition to diabetes and macrosomia include postterm pregnancy, history of shoulder dystocia in a prior pregnancy, maternal obesity, advanced maternal age, platypelloid pelvis, labor induction, and operative vaginal delivery [6]. A recent population-based registry study performed in Norway found that the odds ratio (adjusted for birth weight) for shoulder dystocia in cases of intrauterine fetal demise was 5.9 when compared to live births [9]. Furthermore, the study found that amongst diabetic mothers, the odds ratio for shoulder dystocia in cases of stillbirth was 10.2 when compared to live births.

A number of plausible mechanisms have been proposed to explain the relationship between intrauterine fetal demise and shoulder dystocia. IUFDs are frequently diagnosed prior to the onset of labor, resulting in an induction which is itself a risk factor for shoulder dystocia [10]. Additionally, dilation of pelvic floor muscles occurs in late pregnancy with decrease in muscle tone to promote easier delivery. These changes may not occur in cases of IUFD [11]. Finally, the fetus is known to play an active role in successful delivery [12]. The absence of muscle tone in a fetal demise can therefore also contribute to the increased risk of shoulder dystocia in stillbirth.

Diabetes has been associated with stillbirth and shoulder dystocia. Stillbirth itself is also associated with shoulder dystocia. To date, the data published by the Larsen group in Norway is the only literature available on the triad of poor glycemic control, fetal demise, and shoulder dystocia. Here, we describe two cases of this triad that occurred at Naval Medical Center Portsmouth.

## 2. Case 1

A 34-year-old gravida two, abortus one, para zero presented to the Labor and Delivery Unit at 40 weeks and three days estimated gestational age. She had been diagnosed earlier that day with an intrauterine fetal demise by the certified professional midwife who had been managing her pregnancy. The patient had not been seen by a physician nor a certified nurse midwife throughout the gestation. On review of her records, it was noted that the standard 50-gram one-hour glucose tolerance testing (GTT) had been deferred in favor of one day of monitoring of fasting and postprandial blood glucose levels at approximately 25 weeks gestational age. These values were not available for review but were reportedly normal per the patient. No testing of glycated hemoglobin (hemoglobin A1c) was performed while pregnant, and serial blood glucose monitoring was not repeated after 25 weeks gestational age.

The patient consented to our institution's standard panel of laboratory testing for patients diagnosed with intrauterine fetal demise. This testing includes a complete blood count, prothrombin time, partial thromboplastin time, fibrinogen level, hemoglobin A1c, thyroid stimulating hormone, urine drug screen, Kleihauer-Betke, rapid plasma reagin, parvovirus B19 antibodies, cytomegalovirus antibodies, lupus anticoagulant antibodies, beta-2 glycoprotein antibodies, anticardiolipin antibodies, factor V Leiden mutation, and prothrombin gene mutation G20210A.

The only abnormality from this set of laboratory results was a hemoglobin A1c value of 6.6%, diagnostic for diabetes based on criteria developed by the American Diabetes Association [13]. No prepregnancy hemoglobin A1c or random glucose values were available for review.

The patient underwent an induction of labor with serial doses of misoprostol followed by Pitocin augmentation and artificial rupture of membranes. She met criteria for preeclampsia with severe features during her intrapartum course. She progressed to complete dilation and 0 station after approximately 36 hours. Pushing was initiated shortly thereafter, resulting in expeditious descent of the fetal head to +3 station. No further descent was appreciated over the next hour of pushing. Citing exhaustion, the patient requested assistance via an operative vaginal delivery. Forceps were applied and were successful in delivering the fetal head. The anterior shoulder did not deliver with gentle downward traction.

A shoulder dystocia was identified. McRoberts maneuver with suprapubic pressure did not relieve the dystocia. The Rubin II and Wood's Screw maneuvers were similarly unsuccessful. The posterior arm was successfully delivered, but the shoulder remained impacted. McRoberts maneuver with suprapubic pressure was repeated, followed by the rotational maneuvers again without success.

These maneuvers were not repeated in the delivery room as the fetal head had partially avulsed from the body. The patient was transported to the operating theater due to the possibility of requiring a fragmented delivery. Uterine relaxation was achieved with 100 micrograms of intravenous nitroglycerin. This allowed the anterior shoulder to be disimpacted with Wood's Screw maneuver resulting in the delivery of an infant weighing 4190 grams (85th percentile for gestational age). The total length of the shoulder dystocia was 30 minutes.

## 3. Case 2

A 31-year-old gravida one, para zero, presented to the Labor and Delivery Unit at 37 weeks and one day estimated gestational age with a complaint of decreased fetal movement. She was found to have an intrauterine fetal demise.

On review of her records, it was noted that she had undergone a hemoglobin A1c measurement and 50-gram glucose tolerance test at her first obstetric appointment (nine weeks gestational age) per our institutional protocol due to her elevated prepregnancy body mass index of 34. Her hemoglobin A1c was nondiagnostic (5.0%), but her early GTT returned elevated at 160 mg/dL (Table 1). A follow-up three-hour 100-gram GTT was performed with fasting, one-hour, two-hour, and three-hour values of 86, 184, 147, and 135 mg/dL, respectively. No diagnosis of glucose intolerance was made due to having a single elevated value per the Carpenter Coustan criteria. She was advised to undergo a repeat three-hour GTT at 25 weeks gestational age, but deferred testing until 36 weeks gestational age. Her 36-week 100-gram GTT resulted with fasting, one-hour, two-hour, and three-hour values of 89, 216, 153, and 101 mg/dL, respectively. Again, no diagnosis of glucose intolerance was made due to having a single elevated value.

The patient consented to our institution's standard panel of laboratory testing for patients diagnosed with intrauterine fetal demise as previously described. The only abnormality from this set of laboratory results was a hemoglobin A1c value of 6.6%, diagnostic for diabetes based on criteria developed by the American Diabetes Association.

The patient underwent an induction of labor with serial doses of misoprostol followed by Pitocin augmentation and artificial rupture of membranes. She met criteria for preeclampsia with severe features during her intrapartum course. She progressed to complete dilation after approximately 24 hours. Pushing was initiated shortly thereafter, resulting in the delivery of the fetal head. However, the anterior shoulder did not deliver with gentle downward traction.

A shoulder dystocia was identified. McRoberts maneuver with suprapubic pressure did not relieve the dystocia. The Rubin II and Wood's Screw maneuvers were similarly unsuccessful. The patient was rotated into the Gaskin position but the shoulder remained impacted. The patient was turned back into McRoberts position where suprapubic pressure was again unsuccessful. The rotational maneuvers were also repeated without success. Delivery of the posterior arm was attempted but could not be accomplished. A posterior axilla sling was placed using a pediatric feeding tube. These devices are available in the delivery rooms of our institution as an adjunct in the treatment of intractable shoulder dystocia [14]. Traction on the sling was not successful in delivering the posterior shoulder. The sling was then used in a rotational manner to assist with a repeat attempt at Wood's Screw maneuver as described by Cluver and Hofmeyr [15]. This was successful in relieving the shoulder dystocia resulting in the delivery of an infant weighing 3590 g (92nd percentile for gestational age). The total length of the shoulder dystocia was 30 minutes.

## 4. Discussion

The patient in Case 1 underwent screening that did not meet the standard of care. A single day of fasting and postprandial blood glucose monitoring was performed with reportedly normal results. No glucose tolerance testing was performed. She did not report any objections to undergoing a GTT and stated that the single day of testing was the only screening option offered.

Not all patients are able to tolerate hyperosmolar glucose, so alternative screening schemes have been described such as serial fasting values or periodic testing of hemoglobin A1c [16]. These alternative testing regimens have been found to have sensitivities as low as 88% [17] compared to 99% [18] for glucose tolerance testing. Glucose tolerance testing therefore remains the standard of care.

As per the American Diabetes Association, the hemoglobin A1c value of 6.6% (>6.5%) at diagnosis of term intrauterine fetal demise suggests that her glucose tolerance had been impaired for at least 12 weeks prior to arrival at our facility. It is therefore plausible that a GTT performed at 24-28 weeks gestational age could have identified gestational diabetes in this patient. Had a timely diagnosis of diabetes been made in this patient, daily glucose monitoring, serial growth scans, and nonstress testing would have been recommended. Timing of delivery would have been recommended pending fetal growth and glycemic control.

An additional major risk factor for shoulder dystocia in this case was the use of forceps to effect an operative vaginal delivery. The patient cited emotional and physical exhaustion and expressed an inability to continue pushing after approximately 90 minutes. Despite the increased risk of shoulder dystocia in the setting of impaired glucose tolerance and fetal demise, operative vaginal delivery was offered to obviate the surgical risks of cesarean section, especially without a fetal indication for surgical delivery.

The infant was noted to have a birth weight of 4190 grams. Birth weight greater than 4000 grams is a known sequela of diabetes in pregnancy and is an independent risk factor for shoulder dystocia. Other anthropometric measurements of the infant, such as shoulder-to-head and chest-to-head proportions, were not obtained.

In contrast to Case 1, the patient in Case 2 underwent glucose screening prior to the diagnosis of fetal demise. She was identified as being at risk for pregestational diabetes due to her body mass index and underwent appropriate glucose tolerance testing soon after her intake to obstetric care. Her one-hour GTT was elevated, prompting a 3-hour GTT which was nondiagnostic with one elevated value. Repeat testing was advised as appropriate between 24 and 28 weeks but not performed due to patient factors until 36 weeks. Again, testing was nondiagnostic with a single elevated value though notably a second value was within the normal range by only 2 mg/dL.

The patient's hemoglobin A1c was diagnostic for diabetes at 6.6% on admission for her term intrauterine fetal demise one week later, which demonstrates that even the three-hour GTT with its sensitivity of 99% can fail to diagnose a patient with diabetes. This suggests that the Carpenter Coustan criteria were not sufficiently conservative to diagnose this patient's glucose intolerance. That being said, no guidelines exist regarding monitoring of hemoglobin A1c during pregnancy, and a physiologic decrease in hemoglobin A1c from the first trimester to the third trimester is known to occur [19]. The patient's infant had a birth weight of less than 4000 grams but did meet the criteria for being large for gestational age (LGA) as per the Fenton calculation [20].

Both patients represent cases of fetal demise and protracted shoulder dystocia in the setting of inadequate or ineffective screening for glycemic control in pregnancy. The two patients met the criteria for diabetes via their hemoglobin A1c values on admission for delivery after not meeting the screening criteria for the disease earlier in pregnancy. Both patients were admitted with term fetal demise. While both patients declined autopsies of their infants, neither had any risk factor for stillbirth other than poor glucose control. Likewise, both patients gave birth to macrosomic or LGA infants in deliveries complicated by 30-minute shoulder dystocia.

The Larsen group analyzed the Medical Birth Registry of Norway from 1967 to 2012 and found that the odds ratio for shoulder dystocia in cases of IUFD was 5.9 compared to live births. The study also concluded that amongst diabetic mothers, the odds ratio for shoulder dystocia in cases of stillbirth was 10.2 compared to live births to diabetic mothers. This is the only study in the literature to report on the triad of diabetes, fetal demise, and shoulder dystocia.

Obstetric providers should be prepared to manage protracted shoulder dystocia in cases of fetal demise in patients with a history of diabetes. The two cases presented here represent the first reporting on this tragic triad in the United States.

## Figures and Tables

**Table 1:** Assessment of glycemic control in Case 2.

Weeks gestation	Test	Value	Normal range
9	Hemoglobin A1c	5.0%	<5.7%
	50 g 1-hour GTT	160 mg/dL	<140 mg/dL
	100 g 3-hour GTT	86 mg/dL fasting	<95 mg/dL
		184 mg/dL at 1 hour	<180 mg/dL
		147 mg/dL at 2 hours	<155 mg/dL
		135 mg/dL at 3 hours	<140 mg/dL

36	100 g 3-hour GTT	89 mg/dL fasting	<95 mg/dL
		216 mg/dL at 1 hour	<180 mg/dL
		153 mg/dL at 2 hours	<155 mg/dL
		101 mg/dL at 3 hours	<140 mg/dL

37	Hemoglobin A1c	6.6%	<5.7%

GTT: glucose tolerance test.
